# Synovial explant inflammatory mediator production corresponds to rheumatoid arthritis imaging hallmarks: a cross-sectional study

**DOI:** 10.1186/ar4557

**Published:** 2014-05-05

**Authors:** Martin Andersen, Mikael Boesen, Karen Ellegaard, Robin Christensen, Kalle Söderström, Niels Søe, Pieter Spee, Ulrik GW Mørch, Søren Torp-Pedersen, Else Marie Bartels, Bente Danneskiold-Samsøe, Nina Vendel, Lars Karlsson, Henning Bliddal

**Affiliations:** 1 The Parker Institute, Department of Rheumatology, Copenhagen University Hospital, Bispebjerg and Frederiksberg, Nordre Fasanvej 57, Copenhagen 2000 Frederiksberg, Denmark; 2 Department of Translational Immunology, Biopharmaceutical Research Unit, Novo Nordisk, Novo Nordisk Park 1, 2760 Måløv, Denmark; 3 Department of Radiology, Copenhagen University Hospital, Bispebjerg and Frederiksberg, Bispebjerg Bakke 23, Copenhagen 2400, København NV, Denmark; 4 Department of Orthopedics, Section of Hand Surgery, Gentofte University Hospital, Gentofte Hospital, Niels Andersens Vej 65, 2900 Hellerup, Denmark; 5 Biomarkers, Novo Nordisk, Vandtårnsvej 108, 2860 Søborg, Denmark; 6 Department of Anaesthesiology, Intensive Care and Operations, Gentofte University Hospital, Gentofte Hospital, Niels Andersens Vej 65, 2900 Hellerup, Denmark

## Abstract

**Introduction:**

Despite the widespread use of magnetic resonance imaging (MRI) and Doppler ultrasound for the detection of rheumatoid arthritis (RA) disease activity, little is known regarding the association of imaging-detected activity and synovial pathology. The purpose of this study was to compare site-specific release of inflammatory mediators and evaluate the corresponding anatomical sites by examining colour Doppler ultrasound (CDUS) and MRI scans.

**Methods:**

RA patients were evaluated on the basis of CDUS and 3-T MRI scans and subsequently underwent synovectomy using a needle arthroscopic procedure of the hand joints. The synovial tissue specimens were incubated for 72 hours, and spontaneous release of monocyte chemoattractant protein 1 (MCP-1), interleukin 6 (IL-6), macrophage inflammatory protein 1β (MIP-1β) and IL-8 was measured by performing multiplex immunoassays. Bone marrow oedema (BME), synovitis and erosion scores were estimated on the basis of the rheumatoid arthritis magnetic resonance imaging score (RAMRIS). Mixed models were used for the statistical analyses. Parsimony was achieved by omitting covariates with *P* > 0.1 from the statistical model.

**Results:**

Tissue samples from 58 synovial sites were obtained from 25 patients. MCP-1 was associated with CDUS activity (*P* = 0.009, approximate Spearman’s ρ = 0.41), RAMRIS BME score (*P* = 0.01, approximate Spearman’s ρ = 0.42) and RAMRIS erosion score (*P* = 0.03, approximate Spearman’s ρ = 0.31). IL-6 was associated with RAMRIS synovitis score (*P* = 0.04, approximate Spearman’s ρ = 0.50), BME score (*P* = 0.04, approximate Spearman’s ρ = 0.31) and RAMRIS erosion score (*P* = 0.03, approximate Spearman’s ρ = 0.35). MIP-1β was associated with CDUS activity (*P* = 0.02, approximate Spearman’s ρ = 0.38) and RAMRIS synovitis scores (*P* = 0.02, approximate Spearman’s ρ = 0.63). IL-8 associations with imaging outcome measures did not reach statistical significance.

**Conclusions:**

The association between imaging activity and synovial inflammatory mediators underscores the high sensitivity of CDUS and MRI in the evaluation of RA disease activity. The associations found in our present study have different implications for synovial mediator releases and corresponding imaging signs. For example, MCP-1 and IL-6 were associated with both general inflammation and bone destruction, in contrast to MIP-1β, which was involved solely in general synovitis. The lack of association of IL-8 with synovitis was likely underestimated because of a large proportion of samples above assay detection limits among the patients with the highest synovitis scores.

## Introduction

Rheumatoid arthritis (RA) is a chronic inflammatory disease characterised by progressive joint damage. The aetiology of RA is, to a large extent, unknown, and current therapies are aimed at reducing the inflammatory disease burden. Recent data support the practice of frequent monitoring of disease activity in RA patients to achieve remission faster [1,2]. However, patients may still experience radiographic progression of bone erosions, despite being judged to be in clinical remission [1,3,4]. The emergence of imaging techniques such as ultrasound (US) and magnetic resonance imaging (MRI) have paved the way for detailed descriptions of joint inflammation and erosion. These imaging modalities have recently been accepted as part of the diagnostic criteria in RA [1,5]. Because inflammatory activity detected by MRI or Doppler ultrasound (DUS) has shown to be a good predictor of disease course and response to treatment, the expansion of the use of these detection modalities in clinical assessments of RA has been suggested [6-8].

The invasive front of the synovium—the pannus—is believed to be the main driver of cartilage degradation and bone erosion; however, to the best of our knowledge, no researchers to date have investigated the association of histological features of synovitis with the extent of bone marrow oedema (BME) and erosions visualised by MRI. This information would be interesting because the presence of cluster of differentiation–positive (CD68+) macrophages in RA synovium has been linked to the development of erosions detected on radiographs [9]. Investigators who conducted MRI-based studies focused mainly on, and demonstrated, associations of MRI-detected synovitis and synovial histopathology [10-12] with MRI-detected BME and osseous histopathology [13-15]. Furthermore, previously published data have generally been based on small sample sizes. Also, the synovial material examined in the vast majority of cases was obtained from knee joints of patients undergoing knee joint replacement surgery in cases where inflammation due to concomitant osteoarthritis may have confounded the results. Current evidence is based mainly on studies in which the investigators assessed pathology on the basis of whole-joint imaging, which may have decreased sensitivity for the detection of local activity in the different areas of the synovium.

Studies of RA synovial explants have demonstrated the value of the capacity they provide for evaluating inflammatory output and the response to anti-inflammatory treatment, as well as for visualising the morphologic features of the RA synovium [16-24]. Several methods of establishing synovial cultures have been described, but the use of assays based on whole synovial tissue has been recommended in order to maintain synovial architecture and cell-to-cell contact [25]. Researchers in prior biopsy-based explant studies might not have taken the heterogeneous synovial tissue distribution into account, however, thus compromising the explant assays’ ability to detect overall joint inflammation [18,26]. In recent years, novel immunoassay techniques, such as multiplex technology, have paved the way for simultaneous analysis of multiple cytokines and chemokines in a small sample. However, it has been shown that synovial production of heterophilic antibodies (HAs), such as rheumatoid factor (RF), can have a great impact on assay reliability if they are not blocked [27]. In contrast to synovial biopsies, explants consist of live cells, and the production of inflammatory mediators from these intact tissue cultures may thus provide novel information about RA pathogenesis.

Our aim in this study was to compare the association of synovial inflammatory mediator production with the colour Doppler ultrasound (CDUS) fraction [28] and/or the rheumatoid arthritis magnetic resonance imaging score (RAMRIS) [7] using 3-T MRI scans of the corresponding anatomical sites in the target hand. Obtaining synovial tissue by synovectomy under direct visual control enabled us to harvest as much of the synovium as possible from the different anatomical sites of the hand joints. The results of this study should thus provide novel insights into the association between synovial inflammatory mediators and RA hallmarks.

## Methods

### Patients

Patients with RA [29] and synovial hypertrophy diagnosed on the basis of greyscale US image analysis were eligible for the study. We excluded patients who were under 18 years of age, allergic to local anaesthetics, undergoing anticoagulant treatment or taking a prednisolone dose above 10 mg/day, receiving intraarticular prednisolone injections into the hand, had synovectomy performed within the previous 3 months or had bad skin at the site of interest. Otherwise, no patient selection method was utilised. The enrolment period dated from June 2011 through January 2013. Patient recruitment and clinical and imaging examinations were performed at the outpatient clinic of the Department of Rheumatology at Copenhagen University Hospital, Bispebjerg and Frederiksberg, in Denmark. The study was approved by the local ethics committee of the capital region of Denmark (H-4-2009-117). The approval covered all centres involved in the study, and signed, informed consent was obtained from each patient. Further details about the centres involved are given in the Acknowledgements.

### Imaging modalities

#### *Ultrasound examination*

The evaluation was performed by an experienced US specialist (KE or STP) [31], and CDUS was used to assess the vascularisation of the synovial tissue. The region of interest (ROI) was defined as the synovial tissue. The maximal systolic colour fraction (CF_max_) in the ROI was selected as a marker of synovial inflammation. Colour Doppler mode was chosen as a marker of synovial inflammation because the sensitivity of detecting synovial blood flow achieved with the LOGIQ E9 imaging system (GE Healthcare, Waukesha, WI, USA) was higher than that possible using the power Doppler modality [32]. US examinations were performed as previously described using a 15-MHz center frequency linear array matrix [31]. In brief, the Doppler preset was adjusted for maximum sensitivity at low flow (pulse repetition frequency of 0.4 kHz, lowest wall filter of 45 Hz and 7.5-MHz Doppler frequency), with Doppler gain just below noise level. This preset remained unchanged throughout the study period. In all patients, the wrists, proximal interphalangeal (PIP) and metacarpophalangeal (MCP) joints were examined by US to identify the joints with the most pronounced involvement. All examinations were performed from standardised dorsal and dorsolateral positions using specific anatomic landmarks in the US image in the various positions [33]. Following identification of the anatomic landmarks in the greyscale image, the Doppler was activated and, while keeping the landmarks in the image, the transducer was adjusted until the scan plane with the most Doppler activity was identified. The transducer was held in this position for a couple of heart cycles, whereupon the image was frozen. By using the cineloop function, the frames with maximum and minimum Doppler activity corresponding to the systole were stored and transferred to a processing program. The synovial Doppler activity was calculated as the ratio of the systolic CDUS pixel count per unit of greyscale pixel count, defined as CF_max_[28]. In the wrist, the synovial tissues in the radiocarpal (RC) and midcarpal (MC) joints were evaluated separately if possible. Colour Doppler mode images were chosen to detect markers of synovial inflammation, because its sensitivity in detecting synovial blood flow on the LOGIQ E9 imaging system was higher than that of the power Doppler mode [32].

Ventral scanning positions were omitted because only the dorsal part of the joint was synovectomised. The examiner was blinded to all patient characteristics. A table describing the anatomical landmarks is provided in Additional file 1.

A table with definitions of the anatomical landmarks is provided in Additional file 1.

#### *Magnetic resonance imaging*

MRI scans were evaluated by an experienced radiologist (MB). All patients were examined while in a 3-T MRI scanner (MAGNETOM Verio; Siemens, Erlangen, Germany) using a 16-channel cardiac coil covering the target hand. Patients were supine with their hands alongside the body as previously described [34]. Briefly, pre- and post-contrast-enhanced T1-weighted coronal and axial short tau inverted recovery (STIR) sequences were used to calculate synovitis, BME and erosion RAMRIS scores. The RAMRIS synovitis scores, which range from 0 to 3, correspond to no, low, moderate and severe synovitis, respectively, based on subjective evaluation. We used the RAMRIS BME component to evaluate the extent of BME from 0 to 3, where each step corresponded to a 33% increase in BME. The RAMRIS erosion score ranged from 0 to 10, with each step corresponding to 10% increments in bone area eroded in the anatomy of interest. The synovitis scores were performed at the RC and MC levels in the wrist. MRI-based scores for BME and erosion were averaged according to the anatomic location of the synovectomy or if synovectomy positions had been pooled. MB was blinded to all patient characteristics and US data.

### Procedures

The needle arthroscopic procedures were carried out at Section of Hand Surgery, Department of Orthopedics, at Gentofte Hospital in Hellerup, Denmark. The synovial explant culture assay was conducted at the Section of Translational Immunology in the Biopharmaceutical Research Unit at Novo Nordisk, Måløv, Denmark. Needle arthroscopy was performed within 24 hours after clinical, US and MRI examinations. The joints (up to two per patient) that seemed most inflamed clinically and on CDUS were referred for synovectomy [30]. Portals were established laterally for access to the extensor tendons. The RC part of the wrist joint was found at the level of the scapholunate ligament. The radial, central and ulnar joints were located during synovectomies between the scaphoid bone and the distal radius, lunate bone and distal radius, as well as between the triquetral bone and the ulnar meniscus. The MC part of the wrist joint was detected by using a needle probe 1 cm distal from the transverse line of the RC part of the joint. Synovial tissue was obtained from three compartments (between the scaphoid and trapezoid, capitate and lunate, and triquetral and hamate bones, respectively). For the PIP and MCP joints, the landmarks for the portals were the small concavities in the dorsoulnar and dorsoradial directions for the central slip in the PIP joint and through the sagittal band in the MCP joint. With these portal locations, it was possible to obtain good joint visualisation and sufficient distance between the arthroscope, shaver and joint structures. To match imaging pathology with arthroscopic sampling of the synovium, the surgeon (NS) was not blinded to the patient’s diagnosis, medication or US findings. The US description with localisation of Doppler activity and anatomic landmarks enabled the surgeon to sample synovium from the corresponding anatomical sites. MRI pathology was mapped to the synovial samples by use of the local RAMRIS score component corresponding to the synovectomised area.

### Synovial explant cultures

The synovectomy product was transferred under sterile conditions into 15-ml tubes containing RPMI 1640 and GlutaMAX media (Gibco/Life Technologies, Grand Island, NY, USA), 10% foetal bovine serum, 2% heat-inactivated human serum, 1% penicillin and streptomycin (complete medium (CM)). The synovium-containing tubes were kept on wet ice until the explant culture was established and incubated less than 90 minutes postsurgery. The synovial tissue was centrifuged (4°C) at 1,400 rpm for 7 minutes. The CM supernatant was removed by suction, and synovial wet weight was determined. Synovial tissue was distributed in multiple wells at 2 mg/200 μl of CM in a 96-well Nunc MicroWell plate with Nunclon Delta Surface (Thermo Fisher, Waltham, MA, USA) containing bovine bone slices (IDS DT1BON1000-96; Immunodiagnostic Systems Ltd. Boldon, UK). The synovial explant culture was placed in a sterile incubator at 37°C, 100% humidity and 5% CO_2_ for 72 hours, after which supernatants were collected and stored at <80°C until analysed.

Screening of synovial biomarkers of inflammation and testing for HA interference were performed on explant supernatants from eight study participants. The supernatants were assessed by utilising the Human InflammationMAP multiplex immunoassay (Myriad RBM, Austin, TX, USA), which consists of a combination of three multiplex panels to evaluate a total of 46 mediators. Because of budget limitations, one of the three multiplex panels was selected for the study. That panel consisted of 15 mediators, of which interleukin 6 (IL-6), IL-8, monocyte chemoattractant protein 1 (MCP-1) and macrophage inflammatory protein 1β (MIP-1β) could be measured reproducibly from the explant cultures. Subsequent experiments were focused on these analytes. A complete list of the mediators tested is given in Additional file 2. The significance of HA interference was tested prior to analysis by adding 200 μg/ml HeteroBlock (Omega Biologicals, Bozeman, MT, USA), which previously has been reported to block HA interference [27]. For further validation of abolishment of possible HA interference, supernatants were diluted in six serial dilutions from a twofold to sixty-four-fold dilution. Linearity was preserved for all cytokines upon dilution (*R*^2^ = 0.99; data not shown). Supernatant measurements were carried out at the laboratories of Myriad RBM using the Luminex multiplex platform (R&D Systems, Minneapolis, MN, USA). Samples were transported in dry ice under continuous temperature measurements and access to dry-ice supplements. The average explant mediator production explant culture was used in the statistical analysis. Supernatants were added to 200 μg/ml HeteroBlock and run at a 100-fold dilution (stored at 2-fold dilution and further diluted 50-fold when analysed). The lowest limit of detection for the assay was defined by Myriad RBM as the lowest concentration of an analyte in a sample at which the coefficient of variation of replicate standard samples was 30%. The lowest assay detection limits were 72 pg/ml IL-6, 26 pg/ml IL-8, 240 pg/ml MCP-1 and 366 pg/ml MIP-1β. Plate-to-plate variation was less than 10%. MA had access to all patient data. However, none of the imaging data outcome measures were available at the time the explant cultures were established.

## Statistical analysis

Data were clustered within patients; thus a linear mixed model was applied for the statistical tests in order to prevent double-counting errors with inflated standard errors. As previously described, parsimony in the statistical models was achieved by omitting design variables and covariates from the model if no statistical significance was determined (*P* > 0.1). For model optimisation purposes, square root and logarithmic transformations were applied to achieve an approximate Gaussian distribution of residuals. The Spearman’s ρ estimate was considered important to the overall visual data interpretation. Because of double-counting, these Spearman estimates are referred to as approximate Spearman coefficients [35]. Statistical analyses were calculated using SAS version 9.2 software (SAS Institute, Cary, NC, USA). MA and RC had access to all data.

## Results

### Patient and sample characteristics

**Figure 1:**
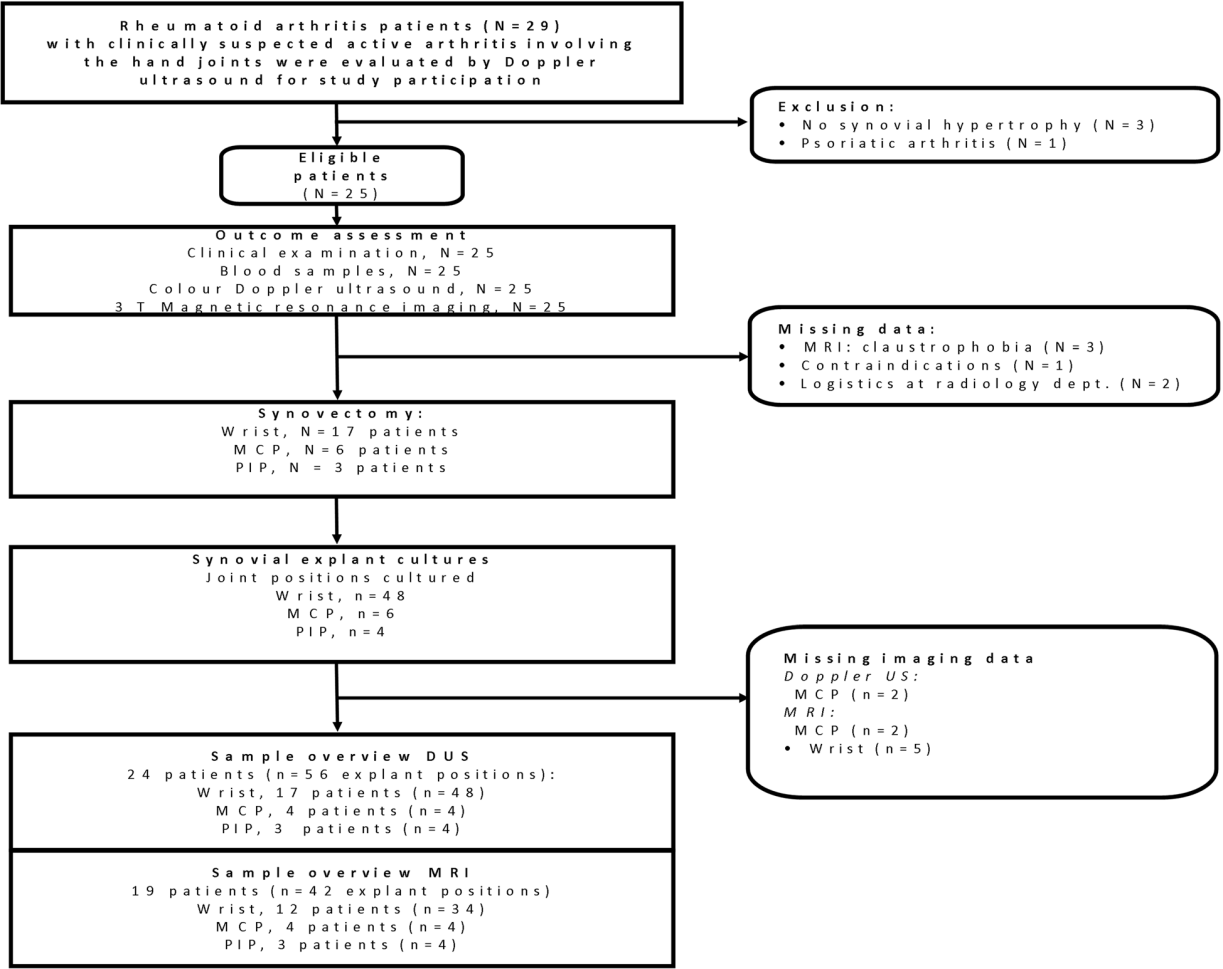
**Schematic of patient flow through the study, imaging procedures carried out, number of joints synovectomised and number of established synovial cultures with corresponding imaging modalities used.** MCP, Metacarpal phalangeal joint; MRI, Magnetic resonance imaging; *N*, Total number of patients; *n*, Number of synovial explant samples; PIP, Proximal interphalangeal joint; US, Ultrasound.

As Figure 1 shows, 25 patients were enrolled into the study, 12 of whom were recruited from a previous study [35]. Three patients who were offered participation declined, and an additional three patients were excluded on the basis of the US examination because of lack of synovial hypertrophy in the hand joints (Figure 1). From among the recruited patients, five (20%) were referred for synovectomy and the remaining twenty (80%) accepted our invitation to participate in the study. The patient population consisted primarily of RF and anticitrullinated peptide antibody (ACPA)–positive women with long-standing disease and mean Disease Activity Score in 28 joints/C-reactive protein (DAS-28-CRP) levels in the upper end of the moderate disease activity interval. The majority (52%) of patients had high disease activity, and 36% had moderate disease activity. Three patients had either low disease activity (*n* = 1) or were classified as in disease remission (*n* = 2) on the basis of DAS28 criteria. Nearly one-third (*n* = 8) were being treated with biological agents (abatacept (*n* = 1), etanercept (*n* = 2), infliximab (*n* = 4) and rituximab (*n* = 1)). Most other patients were being treated with methotrexate as monotherapy or in combination with other disease-modifying antirheumatic drugs (DMARDs) (28% in each category) (Table 1).

**Table 1:** **Patient demographics and clinical characteristics**^**a**^

**Variables**	**Observations (*N *** **= 25)**
Females, *n* (%)	19 (76%)
Mean age ± SD, yr (IQR)	56.2 ± 14.9 (26 to 77)
Disease duration, yr	12.3 [4.0 to 14.1] (0.9; 42.7)
RF-positive, *n* (%)	21 (84%)
Anti-CCP-positive, *n* (%)	22 (88%)
Mean DAS-28-CRP score (0 to 10)	4.8 ± 1.3 [2.2 to 6.8]
CRP, mg/L	20.9 [4 to 30] (1; 117)
VAS global (scale 0 to 100)	62.5 [41 to 83]
Tender joint count, 28 joints	7.1 [4 to 9] (0; 25)
Swollen joint count, 28 joints	7.8 [4 to 9] (1; 21)
DMARD monotherapy	
MTX, *n* (%)	2 (8%)
SZS, *n* (%)	3 (12%)
LFU, *n* (%)	2 (8%)
DMARD combination treatment	
MTX + SZS, *n* (%)	4 (16%)
MTX + SZS + HCQ, *n* (%)	3 (12%)
Biologic DMARDs (%)	8 (32%)
Prednisolone monotherapy, 5 mg/day	2 (8%)
No DMARDs (%)	1 (4%)

^a^CCP, Cyclic citrullinated peptide; CRP, C-reactive protein; DAS28, Disease Activity Score in 28 joints; HCQ, Hydroxychloroquine; LFU, Leflunomide; MCP, Metacarpophalangeal joint; MTX, Methotrexate; PIP, Proximal interphalangeal joint; RF, Rheumatoid factor; SD, Standard deviation; SZS, Sulphasalazine; VAS, Visual Analogue Scale. Values are median [Q1 to Q3], (min; max) or mean ± SD unless otherwise stated.

### Synovial explant mediator production and imaging activity

#### *Synovial explants*

A total of 71 synovectomy specimens were received from 26 joints (17 wrist joints, 6 MCP joints and 3 PIP joints). From among the 71 synovectomies, 58 synovial explant cultures were established. On average, 22 wells were cultured per synovectomy position (SD = 9.4). Three of the joint positions received were used for other purposes, and the remaining ten, due to sparse material, were pooled with one or more joint positions from the same joint. Synovectomy material was pooled from two cases, from two MCP joints in one case and from two PIP joints in the other case. In the cases where synovectomy material was pooled, an average of the imaging data from the corresponding positions was calculated. Three patients had two joints included (that is, two synovectomies), but only one patient with two joints synovectomised had corresponding imaging from both joints (MCP joints).

#### *Magnetic resonance imaging*

Nineteen (forty-two synovectomy positions) of twenty-five patients underwent MRI. As presented in Table 2, the mean RAMRIS BME component score was 2.05 (SD ±0.96), which corresponds to a moderate degree of osteitis ranging from one-third to two-thirds of the bone area. Three patients (seven synovectomies) had no BME. Synovial mediator levels among these patients were above detection limits for MCP-1 (6 (85%) of 7 patients), IL-6 (4 (57%) of 7 patients), IL-8 (7 (100 %) of 7 patients) and MIP-1β (1 (14%) of 7 patients). Among the 16 patients (35 synovectomies) with BME >0, levels of the four measured cytokines were above the assay’s lowest detection limit in 35 (100%), 33 (94%), 35 (100%) and 16 (46%), synovectomies, respectively.

**Table 2:** **Overview of imaging observations and explant activity**^**a**^

**Variable**	**Observations No. patients (No. synovectomies)**	**Median [IQR] (min;max)**
**Imaging**		
Focal RAMRIS BME score, 0-3		
Wrist	12 (34)	1.8 [0.8 to 3.0] (0.0; 3.0)
MCP	4 (4)	2.0 [1.3 to 2.8] (0.0; 3.0)
PIP	3 (4)	2.0 [1.0 to 2.5] (1.0; 2.5)
Total	19 (42)	1.5 [1.0 to 2.8] (0.0; 3.0)
CF_max_, 0-1		
Wrist	17 (48)	0.08 [0.02 to 0.23] (0.0; 0.86)
MCP	4(4)	0.12 [0.05 to 0.34] (0.03; 0.52)
PIP	3(4)	0.06 [0.03 to 0.07](0.0: 0.08)
Total	24 (56)	0.08 [0.01 to 0.21] (0.0; 0.86)
**Explant mediator production, pg/mL**		
MCP-1		
Wrist	17 (48)	4,848 [1,138 to 15,000] (240.5; 46,450)
MCP	6 (6)	2,444 [840 to 11,175] (240.5; 31,336)
PIP	3 (4)	2,082 [986 to 2,707] (348; 3,053)
Total	25 (58)	3,865 [1,138 to 12,915] (240.5; 46,450)
IL-6		
Wrist	17 (48)	8,465 [1,321 to 39469] (72.5; 266,857)
MCP	6 (6)	4,969 [391 to 37,496] (72.5; 534,340)
PIP	3 (4)	11,901 [1,761 to 29,205] (732; 37,400)
Total	25 (58)	8,031 [1,156 to 37,400] (72.5; 534,340)
IL-8		
Wrist	17 (48)	21,129 [5,649 to 96,401] (26,5; 137,001)
MCP	6 (6)	21,132 [5,405 to 48,883] (4,571; 137,001)
PIP	3 (4)	20,730 [6,388 to 50,750] (2,017; 70,800)
Total	25 (58)	21,129 [5,503 to 89,643] (26.5; 137,001)
MIP-1β		
Wrist	17 (48)	1,812 [530 to 6,828] (366.5; 28,420)
MCP	6 (6)	1,078 [577 to 2,786] (366.5; 19,120)
PIP	3 (4)	555 [354 to 1,724] (366.5; 2,705)
Total	25 (58)	1,331 [502 to 5,196] (366.5; 28,420)

Depicting an overview of the imaging activity and synovial explant mediator production. Avr. = average, CF_max_ = colour fraction measured in the systole, IL-6 = Interleukin 6, IL-8 = Interleukin 8, IQR = Interquartile range (3^rd^ quartile – 1^st^ quartile), Max. = maximum, MCP = Metacarpo-phalangeal joint, MCP-1 = macrophage chemoattractant protein 1, Min. = minimum, MIP-1b = Macrophage inflammatory protein 1 beta, no. = number , PIP = Proximal interphalangeal joint, RAMRIS BME Score = Rheumatoid Arthritis Magnetic Ressonance Bone Marrow Oedema Score.

^1^ Mean ± Standard deviation.

#### *Ultrasound*

All 25 patients underwent CDUS. However, the synovectomised joint was not scanned in one patient because of a logistic accident; thus, 24 patients (56 synovectomy positions) were included for the statistical analysis. CDUS scans showed moderate activity on average, with a CF_max_ of 12%. However, 12 patients (48%; 7 with matching CDUS and explant setup) had at least one US position with absence of Doppler activity. Among the 11 synovectomies (11 (20%) of 55) cultured from these Doppler-negative positions, cytokine levels above the lowest detection limit were 9 (81%) of 11 for MCP-1, 4 (64%) of 11 for IL-6, 10 (91%) of 11 for IL-8 and 5 (46%) of 11 for MIP-1β.

### Associations between imaging activity and synovial explant mediator production

**Figure 2:**
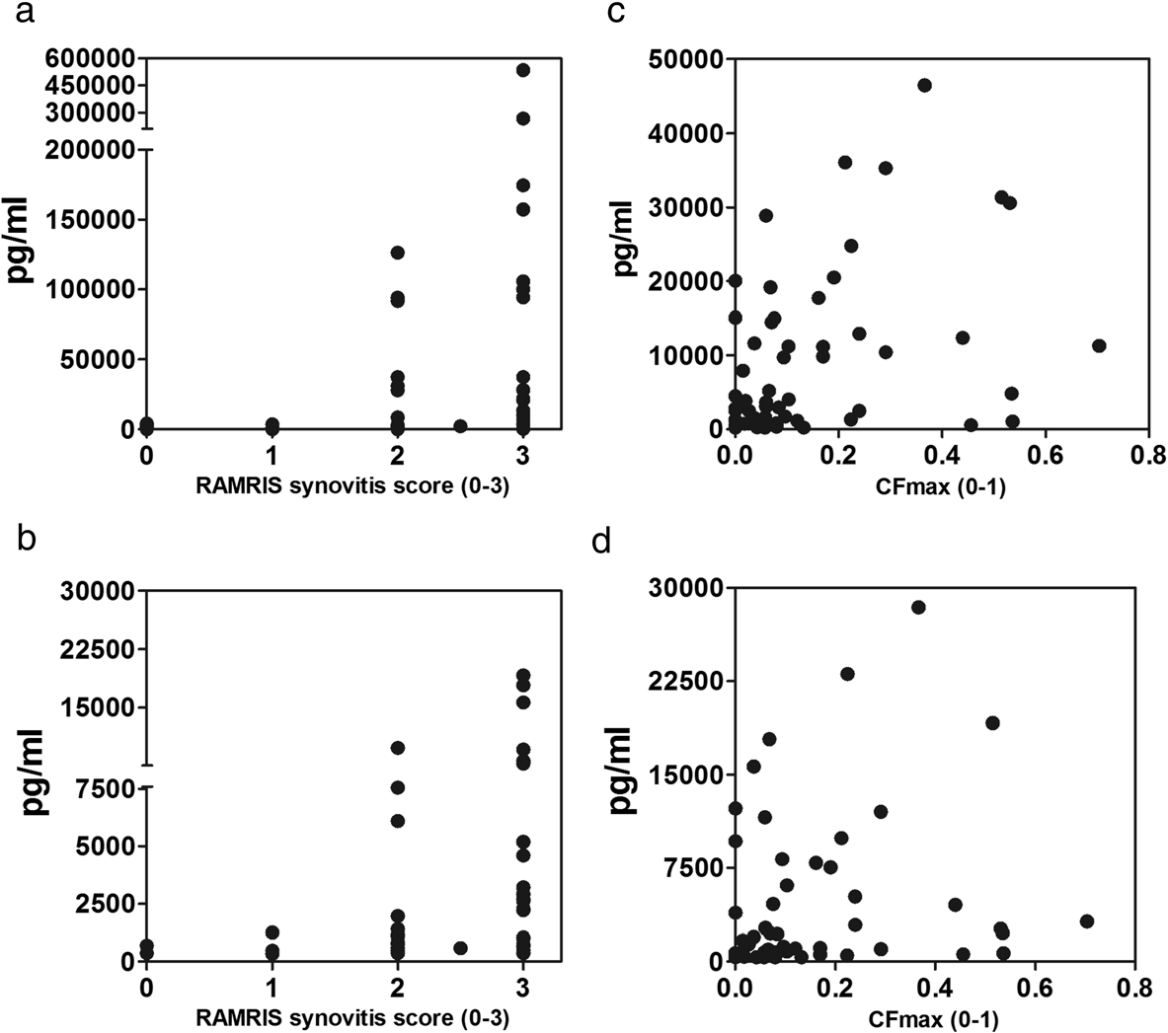
**Scatterplots depicting synovitis, defined as synovial perfusion by maximal systolic colour fraction and by magnetic resonance imaging using the rheumatoid arthritis magnetic resonance imaging score for synovitis vs. synovial explant release after 72 hours in culture.** The statistically significant plots are shown. For the remaining plots, see Additional file 3. Rheumatoid arthritis magnetic resonance imaging scores (RAMRIS) for synovitis are shown in **(a)** and **(b)**. **(a)** Interleukin 6 (*P* = 0.04, approximate ρ = 0.50). **(b)** Macrophage inflammatory protein 1β (*P* = 0.02, approximate ρ =0.63). Maximal systolic colour fraction (CF_max_) values are shown in **(c)** and **(d)**. (**c)** Monocyte chemoattractant protein 1 (*P* = 0.009, approximate ρ = 0.41). (**d)** Macrophage inflammatory protein 1β (*P* = 0.02, approximate ρ = 0.38).

The statistical models show an association between levels of synovial IL-6 (*P* = 0.04, approximated Spearman’s ρ = 0.50) and MIP-1β (*P* = 0.02, approximated Spearman’s ρ = 0.63) and the RAMRIS synovitis score in both the reduced and full statistical models. IL-8 had statistically significant associations in all model reduction steps apart from the final step (*P* = 0.08, approximated Spearman’s ρ = 0.58), whereas MCP-1 did not have a statistically significant association (*P* = 0.17, approximated Spearman’s ρ = 0.48) with regard to the RAMRIS synovitis component score. Synovial perfusion, measured as CF_max_, was associated with levels of MCP-1 (*P* = 0.009, approximated Spearman’s ρ = 0.41) and MIP-1β (*P* = 0.02, approximated Spearman’s ρ = 0.38). In contrast, IL-6 (*P* = 0.34, approximated Spearman’s ρ = 0.22) and IL-8 (*P* = 0.09, approximated Spearman’s ρ = 0.27) were not statistically significantly associated with synovial perfusion. Figure 2 shows scatterplots for the statistically significant associations between RAMRIS synovitis score, CF_max_ and corresponding mediator release. Scatterplots illustrating imaging and explant mediator data not meeting the criteria for statistical significance are provided in Additional files 3 and 4.

Synovial MCP-1 (*P* = 0.01, approximated Spearman’s ρ = 0.42) and IL-6 production (*P* = 0.04, approximated Spearman’s ρ = 0.25) were associated with the RAMRIS BME component score and the RAMRIS erosion component score (*P* = 0.03, approximated Spearman’s ρ = 0.31 for MCP-1) and (*P* = 0.03, approximated Spearman’s ρ = 0.35 for IL-6).

**Figure 3:**
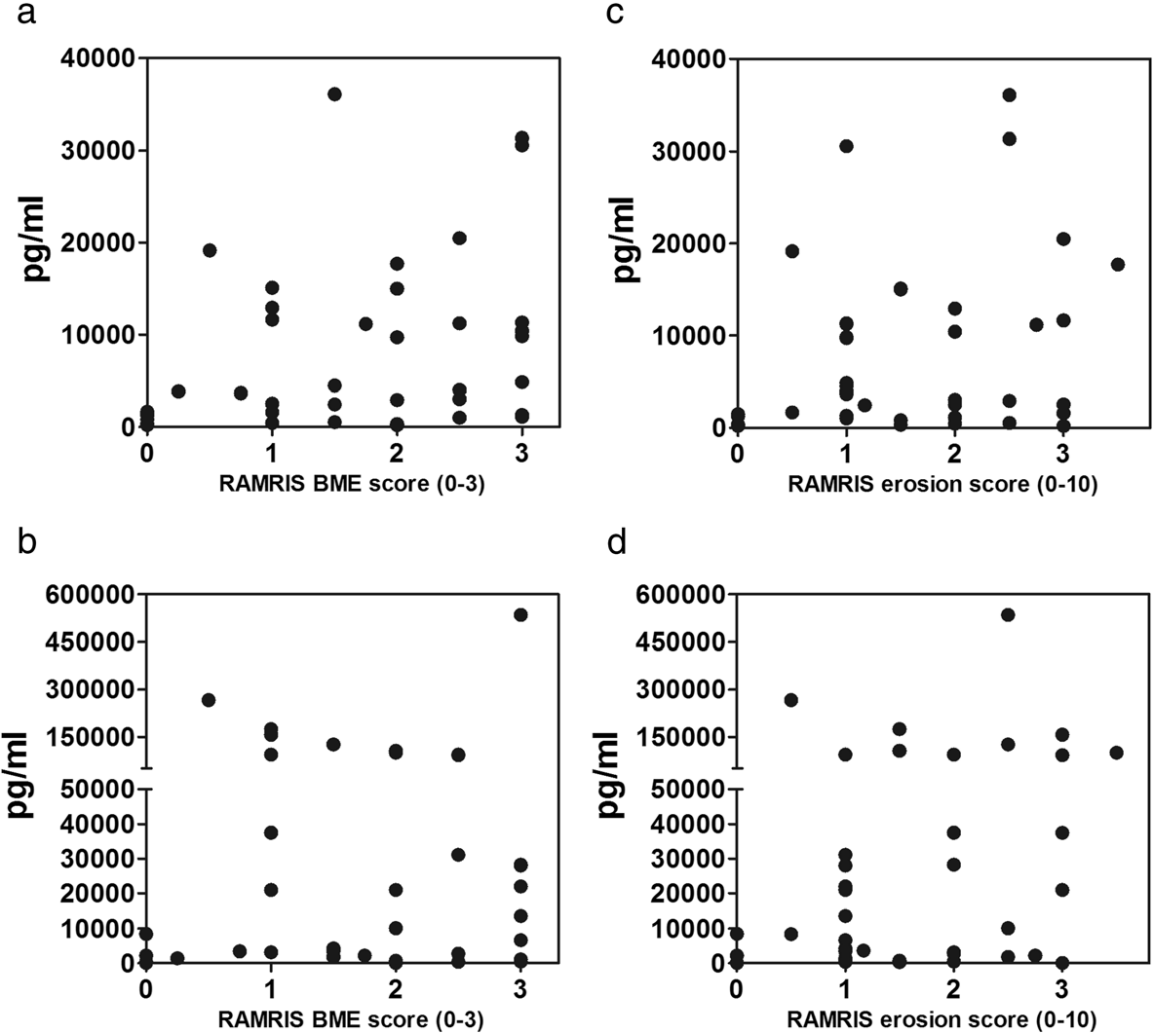
**Scatterplots depicting bone marrow oedema and bone erosion measured by the rheumatoid arthritis magnetic resonance imaging score vs. synovial explant release after 72 hours in culture**. The statistically significant plots are shown. For the remaining plots, see Additional file 4. Bone marrow oedema (BME) rheumatoid arthritis magnetic resonance imaging scores (RAMRIS) are shown in **(a)** and **(b)**. **(a)** Monocyte chemoattractant protein 1 (*P* = 0.01, approximate ρ = 0.42). (B**)** Interleukin 6 (*P* = 0.04, approximate ρ = 0.25). Bone erosion RAMRIS scores are shown in **(c)** and **(d)**. (**c)** Monocyte chemoattractant protein 1 (*P* = 0.03, approximate ρ = 0.31). **(d)** Interleukin 6 (*P* = 0.03, approximate ρ = 0.35).

Table 3 provides a general overview of the findings, including the remaining statistically insignificant findings. Figure 3 shows scatterplots of the data distributions regarding RAMRIS BME and erosion scores and synovial explant release. Additional files 5, 6, 7 and 8 provide details of the statistical model calculations, including the stepwise covariate elimination. All mediators that were statistically significantly associated with CF_max_, RAMRIS synovitis score and RAMRIS BME score were statistically significant in both the unadjusted and adjusted models. With regard to the RAMRIS erosion score, MCP-1 and IL-6 were above the statistical significance level in the unadjusted model (*P* = 0.07 and *P* = 0.08, respectively).

**Table 3:** **Statistical associations with rheumatoid arthritis explant mediator release at 72 hours vs. at time of imaging**^**a**^

**Imaging/mediator**	**CF**_**max**_	**RAMRIS synovitis**	**RAMRIS BME**	**RAMRIS erosion**
	***P*-value (approximate ρ)**	***P*-value (approximate ρ)**	***P*-value (approximate ρ)**	***P*-value (approximate ρ)**
**MCP-1**	0.009 (0.41)	0.17 (0.48)	0.01 (0.42)	0.03 (0.31)
**IL-6**	0.23 (0.22)	0.04 (0.50)	0.04 (0.25)	0.03 (0.35)
**IL-8**	0.09 (0.27)	0.08 (0.58)	0.16 (0.27)	0.07 (0.43)
**MIP-1β**	0.02 (0.38)	0.02 (0.63)	0.95 (0.35)	0.62 (0.30)

^a^CF_max_, Maximal systolic colour fraction; BME, Bone marrow oedema; IL, Interleukin; MCP-1, Monocyte chemoattractant protein 1; MIP-1, Macrophage inflammatory protein 1β; RAMRIS, Rheumatoid arthritis magnetic resonance imaging score.

A mixed model has been used for the statistical analysis: P < 0.05 was considered significant. In the reduced model, covariates were excluded if P > 0.10. This table contains the data from the reduced models. The full models including stepwise covariate elimination steps and model optimization transformation can be found in the supplementary files.

The pre-specified covariates included in the statistical model: Joint Synovectomized = Wrist, MCP or PIP; Synovectomy position = Ulnar, central, radial, or mixed for pooled synovectomy positions; Side = left or right;

Approx. rho = approximated Spearman’s rho; RAMRIS BME = RAMRIS bone marrow edema component; RAMRIS Erosion = RAMRIS erosion score component; RAMRIS synovitis = RAMRIS synovitis score component; IL-6 = Interleukin 6; IL-8 = Interleukin 8, MCP-1 = Monocyte chemoattractant protein 1; MIP-1b = Macrophage Inflammatory Protein 1 beta.

Table 3 presents the statistical associations between RA explant mediator release after 72 hours of culture and imaging activity. Imaging consisted of CDUS activity, reflected as the CF_max_ in the systole. MRI-based activity outcomes were the RAMRIS components. A mixed model was used for the statistical analysis (*P* < 0.05 was considered significant). In the reduced model, covariates were excluded when the *P*-value was >0.10. Table 3 contains the data from the reduced models. The full models, including stepwise covariate elimination steps and model optimisation transformation, are provided in the Additional files. The prespecified covariates included in the statistical model were joints synovectomised (wrist, MCP or PIP), synovectomy position (ulnar, central, radial or mixed for pooled synovectomy positions) and side (left or right).

### Associations between DAS-28-CRP scores and synovial explant mediator production

Ancillary analyses using Spearman’s rank correlations showed that IL-6 (*r* = 0.52, *P* = 0.008), IL-8 (*r* = 0.46, *P* = 0.026) and MCP-1 (*r* = 0.44, *P* = 0.029) were statistically significantly correlated to DAS-28-CRP score. The correlation of MIP-1β to DAS-28-CRP score did not reach statistical significance (*r* = 0.28, *P* = 0.169).

## Discussion

In this study of the hand joints of RA patients, the degree of synovitis—estimated by CDUS using the CF_max_ and by MRI using the RAMRIS criteria—was associated with synovial explant production of key inflammatory mediators. This finding supports previously published observations that DUS and MRI are sensitive imaging modalities for the detection of local inflammation. Among the four mediators investigated in our present study, we observed an interesting polarisation. Whereas IL-6, MCP-1 and MIP-1β were associated with synovitis detected by MRI or CF_max_ in combination, only MCP-1 and IL-6 were associated with the extent of BME and bone erosions visualised by MRI. Furthermore IL-6, IL-8 and MCP-1 production were correlated with DAS-28-CRP score, indicating that the local synovial mediator production reflects systemic disease.

This polarisation in synovial mediator expression is in accord with results produced in biopsy studies in which investigators examined differences in cellular populations and mediator and chemokine receptor expression at the cartilage–pannus junction (CPJ), which is believed to be the site driving the erosive process in the bones of RA patients. In biopsies, IL-6 expression was found exclusively at the CPJ, in contrast to IL-8-positive cells (primarily CD68+ macrophages), the vast majority of which were located at non-CPJ locations [36,37]. The picture is not completely clear, however, because CD68+ macrophages have been linked to radiographically visualised RA progression [38]. MCP-1 and its receptor, chemokine (C-C motif) receptor 2, was located with the highest density near the synovial lining, in contrast to MIP-1β, which had the most pronounced staining in synovial endothelium. MCP-1- and MIP-1β-positive cells, however, were located throughout the synovium [39]. The lack of correlation with DAS-28-CRP score and MIP-1β may indicate that synovial MIP-1β production has a role in RA pathogenesis that is more marginal than that of IL-8, which also had a strong signal (approximate ρ = 0.58) to MRI-detected synovitis. In contrast to MIP-1β, IL-8 was statistically significantly associated with DAS-28-CRP score. However, further studies are needed to verify the clinical significance of the cytokine expression pattern in the synovium.

The lack of association between BME and bone erosion with synovial IL-8 and MIP-1β release can be explained by the main production of these mediators’ originating from non-CPJ areas in the synovium. Researchers in two large clinical studies reported a lack of association of radiographic outcome and synovitis in anti–tumour necrosis factor α (anti-TNF-α)–treated cohorts [40,41]. These findings support our observations of different implications of these mediators in RA pathogenesis and suggest that changes in mediator profiles upon treatment may be able to predict and stratify disease outcomes with regard to synovitis and bone destruction.

Some limitations should be taken into account concerning day-to-day variations in our study. The RA patients in our study had DUS and MRI performed 24 hours prior to surgery, and day-to-day variations may have affected the reported associations [42]. Furthermore, the experimental setup did not include synovectomies from healthy controls. Therefore, baseline information on mediator release from ‘normal’ synovium was not possible. However, cytokine profiling of synovial fluid from healthy controls using a similar Bio-Plex assay setup (Bio-Rad Laboratories, Hercules, CA, USA) showed that the synovial fluid quantities of the four mediators were extremely low, with MCP-1 having the highest mean concentration at 0.5 ± 0.4 pg/ml [30]. We therefore believe that cytokine levels above assay detection limits (lowest detection limit of IL-8 = 26 pg/ml) should reflect synovial pathology. Taking the very low synovial fluid cytokine concentrations into account, the relatively high cutoff values for the assay’s lowest detection limits may have resulted in some overestimation of synovial explant cytokine release. Considering the generally high concentration of synovial explant mediator release, an assay imprecision in the range of 26 to 360 pg/ml, depending on the cytokine, should not affect the overall associations with regard to imaging.

The results of this study show a noteworthy variation in synovial cytokine production and corresponding imaging pathology. The absence of signalling on DUS scans does not rule out synovial cytokine production. These findings are in accord with the results of our previous study in which DUS-detected activity was compared to synovial histopathology in a similar group of RA patients [35]. That previous study did not include grading of the synovial hypertrophy, because the images were obtained only for evaluation of colour Doppler. Further research on the association of synovial hypertrophy with synovial inflammation may provide important insights into RA disease pathogenesis.

Cellular stress inflicted by the *in vitro* circumstances could perhaps have skewed the mediator release from the explant cultures, resulting in higher mediator concentrations. This phenomenon did not generally occur, however, as low concentrations of synovial mediators were observed in some of the explants cultured from sites with moderate to high RAMRIS scores and in the presence of DUS-visualised activity. The observed variations may in fact have been caused by different kinetics controlling synovial perfusion, cellularity and mediator production. These factors may also account for the differences in the cytokines associated with synovial perfusion detected by CF_max_ and with synovitis detected by MRI. Use of biological DMARDs (bDMARDs) may result in changes in cytokine production, but, because of the limited population size in this study, it was not possible to adjust for the different treatment modalities. Further research focused on explants and imaging in a prospective study may clarify the robustness of this method for prognostic use in bDMARD treatment. We made a great effort in designing the study to guide the area of the synovectomy on the basis of the US data; however, a completely accurate match was not possible. MRI mapping of the RAMRIS synovitis score was performed at a regional level in the wrist (RC or MC). Furthermore, BME and bone erosion data were determined as averaged values derived from the synovectomised area. This may represent a source of bias because the synovium was removed only from the dorsal part of the joint. Unfortunately, because of insufficient matching imaging data, a comparison of MRI and CDUS regarding their utility in detecting synovitis was not possible.

The reason IL-8 did not reach a statistically significant association with the MRI synovitis score is likely due to the large number of wells (52%) above the assay’s upper detection limit. None of the other cytokine levels were above the assay detection limit. Among the wells that were above detection levels, an MRI-based synovitis score of 3 was found in 72%, whereas the remaining wells had a score of 2. Budget considerations and the high dilution factors limited the possibilities of including several key inflammatory mediator candidates for measurement, despite their presence in supernatants. These candidate mediators included TNF-α, vascular endothelial growth factor, matrix metalloprotease 3, tissue inhibitor of metalloprotease 1, IL-10 and interferon γ. The synovial explant model therefore offers significant information regarding synovial inflammatory activity. Interestingly, IL-1β levels were very low and under the detection limit (0.57 pg/ml) in five of eight patients in the initial screening. A recent publication also described low levels of IL-1β and TNF-α based on a whole-tissue synovial explant system [43]. This information is in contrast to reports of studies in which enzyme-digested synovial tissue was used, possibly due to changes induced by the digestion process (for example, inhibition of formation of three-dimensional cell layers or lipopolysaccharides in the collagenase, which are potent inducers of IL-1β and TNF-α) [44].

All patients included in our present study had synovial pathology defined by synovial hypertrophy visualised on US scans. Several sites were without DUS-visualised activity, and one case (three synovial explant positions) was judged to have no synovitis on the basis of MRI. Our findings emphasise the importance of considering the degree of greyscale synovitis when evaluating RA patients on the basis of US in the clinic.

## Conclusion

In this study, we show that MRI and CDUS are highly sensitive tools for the detection of synovial pathology. Furthermore, synovial explants can identify imaging biomarkers associated with the RA disease hallmarks synovitis, BME and bone erosions, and the production of several proinflammatory cytokines correlates with overall disease activity, indicating great potential for the use of synovial explant assays in the quest for the identification of novel pathways to aid in understanding RA pathology.

## Abbreviations

BME: Bone marrow oedema; CD68+: Cluster of differentiation 68–positive; CDUS: Colour Doppler ultrasound; CF_max_: Maximal systolic colour fraction; CM: Complete medium; CPJ: Cartilage pannus junction; CRP: C-reactive protein; HA: Heterophilic antibody; HCQ: Hydroxychloroquine; IL: Interleukin; IQR: Interquartile range; LFU: Leflunomide; MC: Midcarpal; MCP: Metacarpal phalangeal; MCP-1: Monocyte chemoattractant protein 1; MIP-1β: Macrophage inflammatory protein 1β; MMP-3: Matrix metalloprotease 3; MRI: Magnetic resonance imaging; MTX: Methotrexate; PIP: Proximal interphalangeal; Q1: First quartile; Q3: Third quartile; RAMRIS: Rheumatoid arthritis magnetic resonance imaging score; RC: Radiocarpal; ROI: Region of interest; SD: Standard deviation; STIR: Short tau inverted recovery; SZS: Sulphasalazine; TIMP-1: Tissue inhibitor of metalloprotease 1; TNF-α: Tumour necrosis factor α; VAS: Visual Analogue Scale; VEGF: Vascular endothelial growth factor.

## Competing interests

During the course of this study Martin Andersen, Kalle Söderstöm, Pieter Spee, Ulrik GW Mørch, and Lars Karlsson were employed at Novo Nordisk. Kalle Söderstöm, Pieter Spee, Ulrik GW Mørch, and Lars Karlsson owned stocks in Novo Nordisk. MA, KS, UGWM, PS and LK were all employed by Novo Nordisk during the patient recruitment period. KS, PS, UGWM and LK owned stocks in Novo Nordisk.

## Authors’ contributors

MA was responsible for the study conception and design, manuscript writing, establishment of the synovial explant assay, data collection and analysis and critical revision of the manuscript. MB was responsible for the study conception and design, manuscript writing, data collection and analysis and critical revision of the manuscript. KE and STP were responsible for the study conception and design, data collection and analysis and critical revision of the manuscript. RC was responsible for the study conception and design, manuscript writing, statistical analyses and critical revision of the manuscript. KS, EMB, NV was responsible for the study conception and design and critical revision of the manuscript. NS was responsible for the study conception and design, manuscript writing, data collection and critical revision of the manuscript. PS, UM, BDS, LK and HB were responsible for the study conception and design, securing funding and critical revision of the manuscript. All authors read and approved the final manuscript.

## Supplementary Material

Additional file 1 **Table depicting the anatomic landmarks defining each ultrasound scan position.** Standardized ultrasound scanning planes, image selection, and image quantification. Description of data: overview of the standardized ultrasound scanning planes and description of the colour Doppler quantification method. Click here for file

Additional file 2 Table giving an overview of the multiplex panel chosen for the supernatant analysis and description of the multiplex panel and rationale behind the selection of synovial mediators used in the study. Click here for file

Additional file 3 **Figure showing scatterplots of statistically insignificant associations of synovial mediator release and CF**_**max **_**and RAMRIS synovitis score.** Scatterplots depict synovitis, defined as synovial perfusion by colour fraction max (CF_max_) and the RAMRIS synovitis score vs. synovial explant release (in pg/ml) after 72 hours of culture that did not reach a statistically significant association. For CF_max_: (a) IL-6 (*P* = 0.23, approximated ρ = 0.22). (b) IL-8 (*P* = 0.09, approximated ρ = 0.27). For the RAMRIS synovitis score: (c) MCP-1 (*P* = 0.17, approximated ρ = 0.48). (d) IL-8 (*P* = 0.05, approximated ρ = 0.58). IL, Interleukin; MCP-1, Monocyte chemoattractant protein 1; RAMRIS, Rheumatoid arthritis magnetic resonance imaging score. Click here for file

Additional file 4 **Figure showing scatterplots of statistically insignificant associations of synovial mediator release and RAMRIS BME and RAMRIS erosion scores.** Scatterplots depict bone erosion measured by the RAMRIS bone marrow oedema (BME) and bone erosion score vs. synovial explant release (pg/ml) after 72 hours in culture that did not reach statistical significance. RAMRIS BME: (a) IL-8 (*P* = 0.16, approximated ρ = 0.27). (b) MIP-1β (*P* = 0.95, approximated ρ = 0.35). RAMRIS erosion: (c) IL-8 (*P* = 0.07, approximated ρ = 0.43). (d) MIP-1β (*P* = 0.62, approximated ρ = 0.30). IL, Interleukin; MIP-1β, Monocyte inflammatory protein 1β; RAMRIS, Rheumatoid arthritis magnetic resonance imaging score. Click here for file

Additional file 5 **Table providing an overview of the stepwise covariate elimination in the statistical models with regard to synovial mediator production and CDUS activity.** This table depicts the statistical associations between colour Doppler ultrasound (p = CDUS) activity and synovial explant mediator release after 72 hours in culture. A mixed model has been used for the statistical analysis. *P* < 0.05 was considered significant. In the reduced model, covariates were excluded if the *P*-value was >0.10. All of the four prespecified covariates tested in the models are shown. Click here for file

Additional file 6 **Table giving an overview of the stepwise covariate elimination in the statistical models with regard to synovial mediator production and RAMRIS synovitis score.** This table depicts the statistical associations between the rheumatoid arthritis magnetic resonance imaging synovitis score (RAMRIS) component in the part of the joint that was synovectomised and synovial explant mediator release after 72 hours in culture. A mixed model was used for the statistical analysis. *P* < 0.05 was considered significant. In the reduced model, covariates were excluded when *P*-values were >0.10. All of the four prespecified covariates tested in the models are shown. Click here for file

Additional file 7 **Table providing an overview of the stepwise covariate elimination in the statistical models with regard to synovial mediator production and RAMRIS BME score.** This table depicts the statistical associations between the rheumatoid arthritis magnetic resonance imaging bone marrow oedema score (focal RAMRIS BME) component and synovial explant mediator release after 72 hours in culture. A mixed model was used for the statistical analysis. *P* < 0.05 was considered significant. In the reduced model, covariates were excluded if *P*-values were >0.10. All of the four prespecified covariates tested in the models are shown. Click here for file

Additional file 8 **Table depicting the statistical associations between the rheumatoid arthritis magnetic resonance imaging erosion score (RAMRIS) component in the part of the joint that was synovectomised and synovial explant mediator release after 72 hours in culture.** Overview of the stepwise covariate elimination in the statistical models with regards to synovial mediator production and the RAMRIS erosion score. A mixed model was used for the statistical analysis. *P* < 0.05 was considered significant. In the reduced model, covariates were excluded if *P* > 0.10. All of the four prespecified covariates tested in the models are shown. Click here for file
