# Supplementary material for: Synovial explant inflammatory mediator production corresponds to rheumatoid arthritis imaging hallmarks: a cross-sectional study
Source: Arthritis Res Ther. 2014 May 5;16(3):R107. doi: 10.1186/ar4557 (PMC4078218; doi:10.1186/ar4557)
Supplement: Additional file 1 — Table depicting the anatomic landmarks defining each ultrasound scan position. Standardized ultrasound scanning planes, image selection, and image quantification. Description of data: overview of the standardized ultrasound scanning planes and description of the colour Doppler quantification method. [file ar4557-S1.docx]

**Additional file 1. Standardised scanning planes, image selection, and image quantification**

| **Position** | **Landmarks** |
| --- | --- |
| Wrist dorsal central | The third extensor digitorum tendon, tip of radius, carpo-metacarpal joint, ossis lunatum and os capitatum. |
| Wrist dorsal radial | The extensor carpi radialis longus tendon, tip of radius, carpo-metacarpal joint, ossis scaphoideum and ossis trapezoideum |
| Wrist dorsal ulnar | The middle of caput ulnae placed minimum 1 cm from the right side and minimum 2 from the left side of the image margins |
| MCP dorsal  Central, radial, ulnar | Joint space, diaphysis of the metarcarpal and proximal phalanx bones. The joint space has to be placed minimum 1½ cm from the right side and minimum 1 cm. from the left side of the image margins |
| PIP dorsal  Central, radial, ulnar | Joint space, diaphysis of the proximal and intermedialt phalanx bones. The joint space has to be placed minimum 1 cm from the right side and minimum ½ cm from the left side of the image margins |
|  |  |

This table depicts the anatomic landmarks defining each ultrasound scan position. MCP = metacarpophalangeal joint, PIP = proximal interphalangeal joint.

CDUS image selection and quantification:

Following identification of the anatomic landmarks in the gray-scale image, the Doppler was activated and, while keeping the landmarks in the image, the transducer was adjusted until the scan plane with the most Doppler activity was identified. The transducer was held in this position for a couple of heart cycles, where upon the image was frozen. With the cine-loop function, the frames with maximum and minimum Doppler activity, corresponding to the systole was stored in DICOM format. The digitally stored images were transferred to a processing program (ImagePro®). The synovial Doppler activity was calculated as the ratio of the systolic CDUS pixel count per unit of gray scale pixel count, defined as CFmax21. In the wrist joint the synovial tissue in the radio-carpal (RC) and mid-carpal (MC) was evaluated separately, if possible. Colour Doppler mode was chosen as a marker of synovial inflammation, since sensitivity of detecting synovial blood flow on the GE Loqic E9 (Milwaukee , Wisconsin , USA ) was higher compared to the power Doppler mode[25]
