# Supplementary figures and images for: Synovial explant inflammatory mediator production corresponds to rheumatoid arthritis imaging hallmarks: a cross-sectional study
Source: Arthritis Res Ther. 2014 May 5;16(3):R107. doi: 10.1186/ar4557 (PMC4078218; doi:10.1186/ar4557)

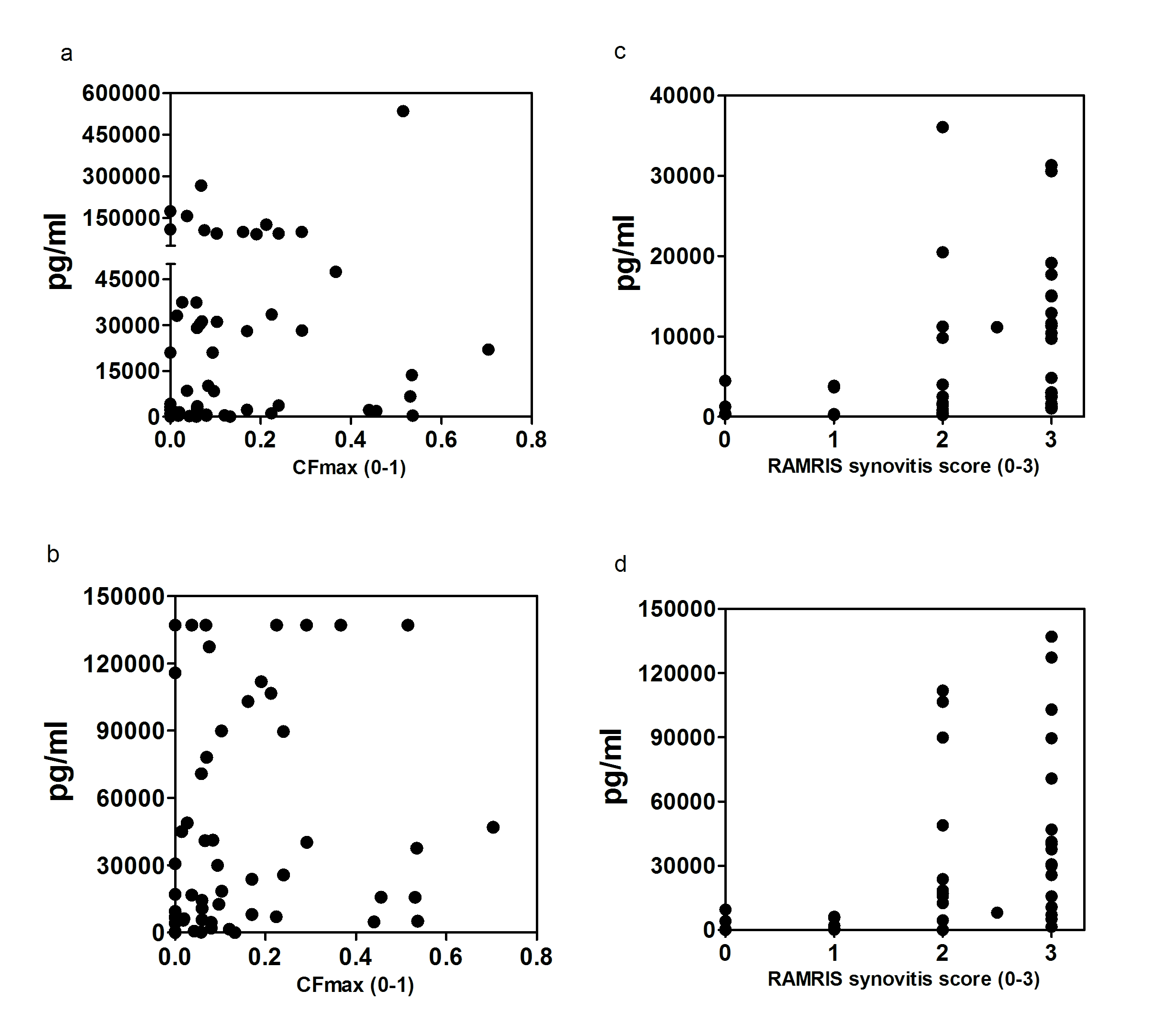

Supplement: Additional file 3 — Figure showing scatterplots of statistically insignificant associations of synovial mediator release and CFmax and RAMRIS synovitis score. Scatterplots depict synovitis, defined as synovial perfusion by colour fraction max (CFmax) and the RAMRIS synovitis score vs. synovial explant release (in pg/ml) after 72 hours of culture that did not reach a statistically significant association. For CFmax: (a) IL-6 (P = 0.23, approximated ρ = 0.22). (b) IL-8 (P = 0.09, approximated ρ = 0.27). For the RAMRIS synovitis score: (c) MCP-1 (P = 0.17, approximated ρ = 0.48). (d) IL-8 (P = 0.05, approximated ρ = 0.58). IL, Interleukin; MCP-1, Monocyte chemoattractant protein 1; RAMRIS, Rheumatoid arthritis magnetic resonance imaging score. [file ar4557-S3.tiff]

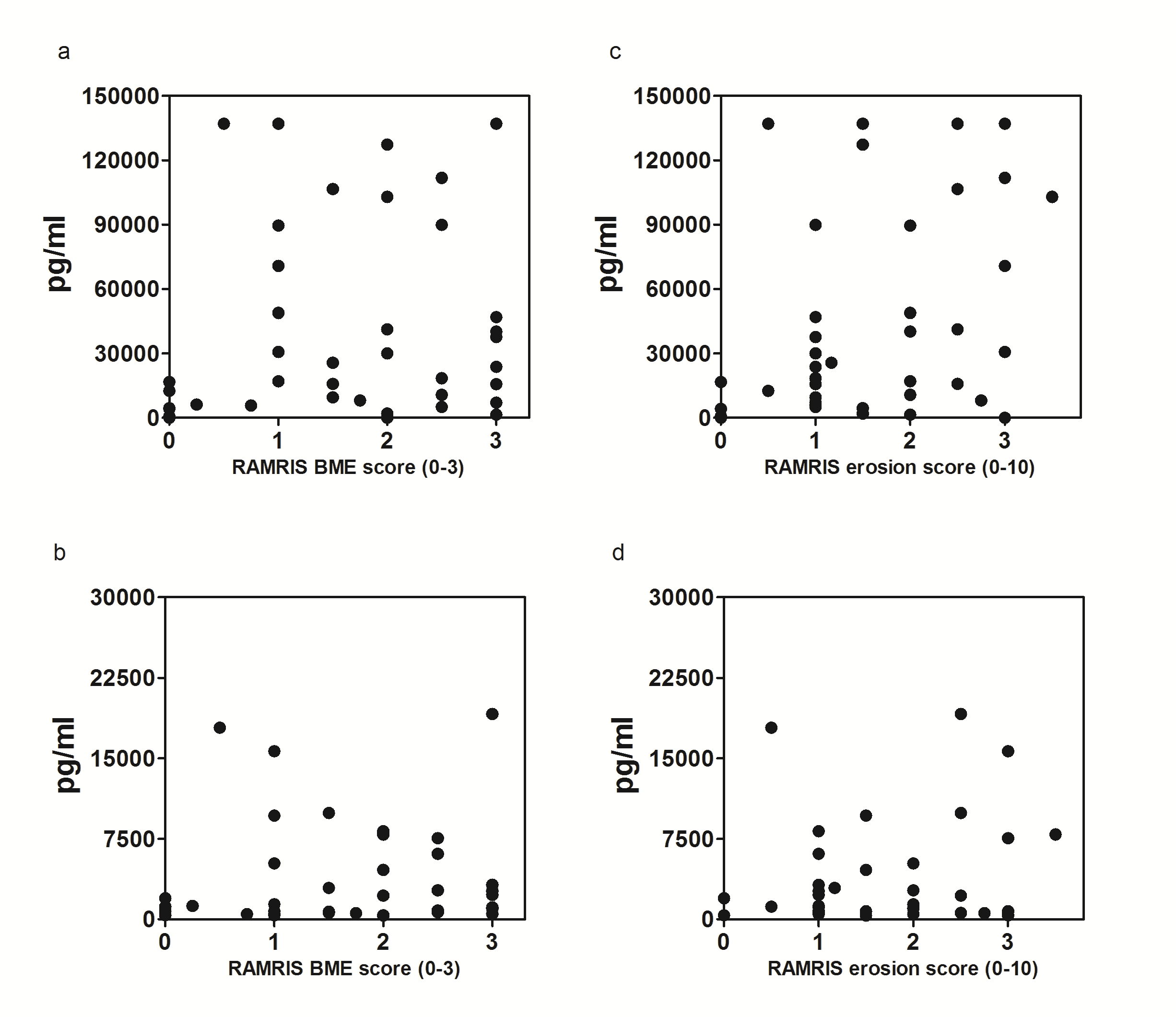

Supplement: Additional file 4 — Figure showing scatterplots of statistically insignificant associations of synovial mediator release and RAMRIS BME and RAMRIS erosion scores. Scatterplots depict bone erosion measured by the RAMRIS bone marrow oedema (BME) and bone erosion score vs. synovial explant release (pg/ml) after 72 hours in culture that did not reach statistical significance. RAMRIS BME: (a) IL-8 (P = 0.16, approximated ρ = 0.27). (b) MIP-1β (P = 0.95, approximated ρ = 0.35). RAMRIS erosion: (c) IL-8 (P = 0.07, approximated ρ = 0.43). (d) MIP-1β (P = 0.62, approximated ρ = 0.30). IL, Interleukin; MIP-1β, Monocyte inflammatory protein 1β; RAMRIS, Rheumatoid arthritis magnetic resonance imaging score. [file ar4557-S4.tiff]
