# Supplementary material for: Synovial explant inflammatory mediator production corresponds to rheumatoid arthritis imaging hallmarks: a cross-sectional study
Source: Arthritis Res Ther. 2014 May 5;16(3):R107. doi: 10.1186/ar4557 (PMC4078218; doi:10.1186/ar4557)
Supplement: Additional file 6 — Table giving an overview of the stepwise covariate elimination in the statistical models with regard to synovial mediator production and RAMRIS synovitis score. This table depicts the statistical associations between the rheumatoid arthritis magnetic resonance imaging synovitis score (RAMRIS) component in the part of the joint that was synovectomised and synovial explant mediator release after 72 hours in culture. A mixed model was used for the statistical analysis. P < 0.05 was considered significant. In the reduced model, covariates were excluded when P-values were >0.10. All of the four prespecified covariates tested in the models are shown. [file ar4557-S6.doc]

**Additional File 6. RA explant mediator release at 72h vs. MRI-activity. Stepwise covariate elimination**

| **Dependent variable** | **Full model**  **(p-value)** | **1^st^ Reduced model (p-value )** | **2nd Reduced model (p-value )** | **3rd Reduced model (p-value )** |
| --- | --- | --- | --- | --- |
| **Log_10_(MCP-1)** | Joint Synovectomized  (p=0.40) |  |  |  |
| **(Approx. Spearman:**  **Rho=0.48)**  **N=19, obs. = 42** | Synovectomy position  (p=0.11) | Synovectomy position  (p=0.10) | Synovectomy position  (p=0.11) |  |
|  | Side  (p=0.31) | Side  (p=0.33) |  |  |
|  | MRI synovitis  (p=0.12) | MRI synovitis  (p=0.13) | MRI synovitis  (p=0.17) |  |
| **Log_10_(IL-6)** | Joint Synovectomized  (p=0.69) |  |  |  |
| **(p=Approx. Spearman:**  **Rho=0.50)**  **N=19, obs.=42** | Synovectomy position  (p=0.34) | Synovectomy position  (p=0.32) |  |  |
|  | Side  (p=0.14) | Side  (p=0.16) | Side  (p=0.17) |  |
|  | MRI synovitis  (p=0.03) | MRI synovitis  (p=0.02) | MRI synovitis  (p=0.02) | MRI synovitis  (p=0.04) |
| **√IL-8** | Joint Synovectomized  (p=0.66) |  |  |  |
| **(Approx. Spearman:**  **Rho=0.58)**  **N=19, obs.=42** | Synovectomy position  (p=0.26) | Synovectomy position  (p=0.24) |  |  |
|  | Side  (p=0.20) | Side  (p=0.22) | Side  (p=0.23) |  |
|  | MRI synovitis  (p=0.05) | MRI synovitis  (p=0.03) | MRI synovitis  (p=0.05) | MRI synovitis  (p=0.08) |
| **Log_10_(MIP-1b)** | Joint Synovectomized  (p=0.53) |  |  |  |
| **(Approx. Spearman:**  **Rho=0.63)**  **N=19, obs.=42** | Synovectomy position  (p=0.08) | Synovectomy position  (p=0.08) | Synovectomy position  (p=0.08) |  |
|  | Side  (p=0.38) | Side  (p=0.37) |  |  |
|  | √MRI synovitis  (p=0.02) | √MRI synovitis  (p=0.02) | √MRI synovitis  (p=0.02) |  |
|  |  |  |  |  |

This table depicts the statistical associations between the rheumatoid arthritis magnetic resonance imaging synovitis score (RAMRIS) component in the part of the joint that was Synovectomized and synovial explant mediator release after 72h of culture. A mixed model has been used for the statistical analysis, P<0.05 was considered significant. In the reduced model covariates were excluded if P>0.10. All of the four pre-specified covariates, tested in the models, are illustrated above.

MRI synovitis = RAMRIS synovitis score, Log_10_ = 10 logarithm, √ = square root.

Covariates included in the statistical model: Joint Synovectomized = Wrist, MCP or PIP; Synovectomy position = Ulnar, central, radial or mixed for pooled synovectomy positions; Side = left or right; IL-6 = Interleukin 6; IL-8 = Interleukin 8; MCP-1 = Monocyte Chemoattractant Protein 1; MIP-1b = Macrophage Inflammatory Protein 1 beta.
