# Supplementary material for: Synovial explant inflammatory mediator production corresponds to rheumatoid arthritis imaging hallmarks: a cross-sectional study
Source: Arthritis Res Ther. 2014 May 5;16(3):R107. doi: 10.1186/ar4557 (PMC4078218; doi:10.1186/ar4557)
Supplement: Additional file 7 — Table providing an overview of the stepwise covariate elimination in the statistical models with regard to synovial mediator production and RAMRIS BME score. This table depicts the statistical associations between the rheumatoid arthritis magnetic resonance imaging bone marrow oedema score (focal RAMRIS BME) component and synovial explant mediator release after 72 hours in culture. A mixed model was used for the statistical analysis. P < 0.05 was considered significant. In the reduced model, covariates were excluded if P-values were >0.10. All of the four prespecified covariates tested in the models are shown. [file ar4557-S7.doc]

**Additional file 7 Table S3. RA explant mediator release at 72h vs. MRI-activity. Stepwise covariate elimination**

| **Dependent variable** | **Full model**  **(p-value)** | **1^st^ Reduced model (p-value )** | **2nd Reduced model (p-value )** | **3rd Reduced model ( p-value )** |
| --- | --- | --- | --- | --- |
| **Log_10_(MCP-1)** | Joint Synovectomized  (p=0.25) |  |  |  |
| **(Approx. Spearman:**  **Rho=0.42)** | Synovectomy position  (p=0.08) | Synovectomy position  (p=0.08) | Synovectomy position  (p=0.09) |  |
| **N=19, obs.=42** | Side  (p=0.10) | Side  (p=0.13) |  |  |
|  | √Focal RAMRIS BME  (p=0.003) | √Focal RAMRIS BME  (p=0.004) | √Focal RAMRIS BME  (p=0.01) |  |
| **Log_10_(IL-6)** | Joint Synovectomized  (p=0.61) |  |  |  |
| **(Approx. Spearman:**  **Rho=0.25)** | Synovectomy position  (p=0.32) | Synovectomy position  (p=0.28) |  |  |
| **N=19, obs.=42** | Side  (p=0.11) | Side  (p=0.13) | Side  (p=0.14) |  |
|  | √Focal RAMRIS BME  (p=0.03) | √Focal RAMRIS BME  (p=0.03) | √Focal RAMRIS BME  (p=0.02) | √Focal RAMRIS BME  (p=0.04) |
| **√IL-8** | Joint Synovectomized  (p=0.63) |  |  |  |
| **(Approx. Spearman:**  **Rho=0.27)** | Synovectomy position  (p=0.36) | Synovectomy position  (p=0.33) |  |  |
| **N=19, obs.=42** | Side  (p=0.19) | Side  (p=0.23) | Side  (p=0.22) |  |
|  | √Focal RAMRIS BME  (p=0.16) | √Focal RAMRIS BME  (p=0.16) | √Focal RAMRIS BME  (p=0.09) | √Focal RAMRIS BME  (p=0.16) |
| **MIP-1b** | Joint Synovectomized  (p=0.40) |  |  |  |
| **(Approx. Spearman:**  **Rho=0.31)** | Synovectomy position  (p=0.05) |  |  |  |
| **N=19, obs. 42** | Side  (p=0.68) |  |  |  |
|  | √Focal RAMRIS BME  (p=0.95) |  |  |  |
|  |  |  |  |  |

This table depicts the statistical associations between the rheumatoid arthritis magnetic resonance imaging bone marrow oedema score ( Focal RAMRIS BME) component and synovial explant mediator release after 72h of culture. A mixed model has been used for the statistical analysis, P<0.05 was considered significant. In the reduced model covariates were excluded if P>0.10. All of the four pre-specified covariates, tested in the models, are illustrated above. √ = Square root. Log_10_= 10 logarithm. N/A= Not available due to hessian criteria not definite positive.

Covariates included in the statistical model: Joint Synovectomized = Wrist, MCP or PIP; Synovectomy position = Ulnar, central, radial or mixed for pooled synovectomy positions; Side = left or right; IL-6 = Interleukin 6; IL-8 = Interleukin 8; MCP-1 = Monocyte Chemoattractant Protein 1; MIP-1b = Macrophage Inflammatory Protein 1 beta.
